# LncRNA PVT1 Mediates Antiapoptosis and 5-Fluorouracil Resistance via Increasing Bcl2 Expression in Gastric Cancer

**DOI:** 10.1155/2019/9325407

**Published:** 2019-05-08

**Authors:** Peizhun Du, Cheng'en Hu, Yunyun Qin, Jing Zhao, Rajan Patel, Yan Fu, Mengqi Zhu, Wenhong Zhang, Guangjian Huang

**Affiliations:** ^1^Department of General Surgery, Huashan Hospital, Fudan University, Shanghai, China; ^2^Primary Care, 8701 Deanna Dr, Gaithersburg, MD 20882, USA; ^3^Department of Infectious Diseases, Huashan Hospital, Fudan University, Shanghai, China

## Abstract

**Purpose:**

Plasmacytoma variant translocation 1 (PVT1) is a long noncoding RNA encoded by the human PVT1 gene, which has been verified to mediate tumorigenesis in gastric cancer. However, the underlying molecular mechanisms of PVT1 in gastric cancer (GC) remain largely unknown.

**Methods:**

The tumorigenic ability of PVT1 was verified by subcutaneous and orthotopic mouse models. Flow cytometry assay and TdT-mediated dUTP Nick-End Labeling staining were conducted to explore the effects of PVT1 on gastric cancer cell apoptosis. We investigated the relative gene and protein that are involved in apoptosis in real-time PCR and western blot assay. The resistance to 5- Fluorouracil (5-Fu) caused by PVT1 was evaluated using cell viability assay. Then, to confirm the effects of PVT1 on 5-Fu resistance, we conducted the Kaplan-Meier analysis based on three public databases.

**Results:**

We confirmed that PVT1 can promote the progression of gastric cancer. PVT1 inhibited the apoptosis of GC cells, which may account for its promotion on GC. We confirmed that PVT1 can regulate the expression of Bcl2 and enhance drug-resistance of gastric cancer to 5-Fu. Kaplan-Meier analysis showed that patients with high PVT1 expression do not experience survival related benefits from 5-Fu based chemotherapy; instead, therapy containing no 5-Fu chemotherapy can improve the first progression survival and overall survival of high PVT1 expression GC patients significantly.

**Conclusion:**

Our results showed that PVT1 can inhibit the apoptosis and enhance the 5-Fu resistance of gastric cancer through the activation of Bcl2. PVT1 has the potential to serve as an indicator to predict 5-Fu treatment resistance.

## 1. Introduction

Gastric cancer (GC) is the second common cancer and the third most common cause of cancer death worldwide [1]. Although radical surgery and perioperative chemotherapy can improve survival of GC patients, the overall survival rate of advanced GC is still less than one year [2]. Many aberrantly expressed genes in GC have been explored in the past decades, but novel molecular markers that can be useful in early diagnosis and treatment of GC are still urgently needed. New therapeutic methods are likely to derive from the improved understanding of the mechanisms of GC.

Formerly, the exploration of mechanisms of malignant tumors was mainly focused on protein-coding genes. Recently, the function of long noncoding RNA (lncRNA) in malignant tumors has attracted increasing attention. LncRNA is identified as the noncoding RNA, which is longer than 200 nucleotides and has limited protein-coding ability. Because of the lack of ability to encode protein, lncRNA was regarded as evolutionary “junk” or “transcriptional noise” in transcription at the beginning stage. But with deepening of exploration, many crucial functionalities of lncRNA in physiological and pathological processes, such as chromatin modification, transcription, and posttranscriptional processing, were revealed. The dysregulation expressed with lncRNA has been demonstrated in multiple malignancies, which provides new insight into the cancer development.

The plasmacytoma variant translocation 1 (PVT1) gene located on chromosome 8q24 is among the top targets of copy number alteration in cancer [3]. An increasing number of studies indicate that lncRNA PVT1 has carcinogenic potential in a variety of tumors. In colorectal cancer cells, silencing PVT1 can decrease proliferation and invasion capabilities by activating TGF–*β* [4]. In hepatocellular carcinoma, PVT1 can promote proliferation and stem cell-like properties of cells by stabilizing NOP2 [5]. In pancreatic cancer, lncRNAPVT1 can promote cell proliferation and migration through acting as a molecular sponge to regulate miR‐448 [6]. Similarly, in gastric cancer, high PVT1 expression is always associated with a poor prognosis. It was reported that PVT1 can function as a competing endogenous RNA by sponging miR-186 [7] and miR-152 [8]. It can also directly bind the FOXM1 protein and increase FOXM1 posttranslationally and epigenetically [9] and can regulate p15 and p16 [10]. However, the current understanding of PVT1 in GC is still in its infancy, and the previous investigations mainly focused on its tumorigenic mechanism in proliferation. Hence the role of PVT1 in other biological process is worth further exploring.

Apoptosis is programmed cell death, which is essential for development and survival of living organisms. It regulates the number of cells by controlling cell activity, differentiation, and proliferation. The defects in apoptotic pathways are now thought to contribute to tumor initiation, progression, and metastasis. What is more, it is well-known now that anticancer agents induce apoptosis and that the dysregulation in apoptotic process can lead to drug-resistance [11]. Biological function of PVT1 in regulating GC apoptosis have been mentioned in relative studies [10, 12], but the internal mechanism of this process is still largely unknown. In the present study, we confirmed PVT1 can inhibit the apoptosis of GC through activating the antiapoptosis factor B cell leukemia 2(Bcl2). This dysfunction in apoptosis caused by PVT1 increased the resistance of GC to anticancer agent 5-Fluorouracil (5-Fu) and made PVT1 a potential reference point in formulating individualized treatment plans.

## 2. Materials and Methods

### 2.1. Cell Culture

Human GC cell lines SGC-7901 was purchased from the Chinese Academy of Sciences (Shanghai, China). It was maintained in RPMI-1640 (Invitrogen, 22400089) medium with 10% fetal bovine serum and 100 u/ml penicillin and 100 ug/ml streptomycin sulphate. The cell was cultured in a humidified 5% CO_2_ at 37°C.

### 2.2. Quantitative Real-Time Reverse Transcription

Trizol reagent was used to extract total RNA. The reverse transcription and real-time PCR (RT-PCR) were conducted by using PrimeScript™ RT reagent kit (TaKaRa, RR037A) and SYBR Premix EX Taq™ II kit (TaKaRa, RR820A) kit, respectively. RT-PCR was implemented under the ABI PRISM 7500 HT Sequence Detection System. GAPDH was adopted as an internal control in RT-PCR to standardize the variants among the different samples. The 2^-ΔΔct^ of RNAs were calculated by premier sequences:

  PVT1-RT-F: CCTGTGACCTGTGGAGACAC;
  PVT1-RT-R: GTCCGTCCAGAGTGCTGAAA;
  Bcl2-RT-F: GGAGGCTGGGATGCCTTTGT;
  Bcl2-RT-R: AAAGCCAGCTTCCCCAATGA;
  GAPDH-RT-F: TCGACAGTCAGCCGCATCTTCTTT;
  GAPDH-RT-R: ACCAAATCCGTTGACTCCGACCTT.

Other premier sequences are listed in Table S1.

### 2.3. Western Blotting Assay

Total protein was obtained from GC cell lines. The total protein was lysated using RIPA with PMSF and quantified using the BCA method. After electrophoretically separated on SDS-PAGE (Sangon Biotech, SD6013), the protein was electrophoresed in 10% or 12% ploy-acrylamide gel. After transferring the protein onto the NC membrane, we blocked the membrane with 5% BSA and incubated it in 1:1000 diluted antibodies against Bcl2 (#2870, CST, USA), Bax (#5023, CST, USA) Caspase 3 (#9665, CST, USA), and *β*-actin (#12620, CST, USA) at 4°C. Secondary antibody was detected using the Odyssey system.

### 2.4. Apoptosis Detection Assay

Apoptosis detection was conducted by using the FITC Annexin V Apoptosis Detection Kit I (BD Pharmingen, 556547). After digesting the GC cell with trypsin, the isolated cell was incubated in Annexin V-FITC, an early-stage apoptosis indicator, and then in propidium iodide, a late-stage apoptosis indicator. The percent of apoptotic cells were detected by cytometry using the BD FACS CantoTM Flow Cytometer.

### 2.5. Plate Clone Formation Assay

Trypsin-digested SGC-7901 cells were seeded into 6-wells plates with 1.5×10^3^cells. Evenly dispersed cell were incubated at 37°C with 5% CO2 until the visible clones appeared. Next, we discarded the medium and washed the cells with PBS twice. We stained the cells with Gentian violet solution after fixing it with methanol. Then we washed the cells and calculated the clone with an ordinary optical microscope.

### 2.6. Experimental Animals

Briefly, the 3-4-week-old 20g Nod/SCID mice were kept in the SPF animal laboratory. Trypsin-digested SGC-7901 cells were inoculated in subcutaneous tissue of the back or stomach to establish subcutaneous or orthotopic models. Each site was injected with 1×10^7^ cells. The subcutaneous xenograft grew for 1 month, and the orthotopic xenograft grew for 2 months, then the mice were satisfied by anaesthesia. To compare the tumorigenicity of GC, the subcutaneous tumors of different groups were measured for diameter. All experiments were performed in accordance with the National Institutes of Health guidelines (NIH Publications No. 8023).

### 2.7. Immunohistochemistry (IHC) and TdT-Mediated dUTP Nick-End Labeling (TUNEL) Assay

All the GC tissue sections were fixed in 4% formalin overnight and embedded in paraffin with standard techniques. The immunohistochemistry detection was conducted by using a SABC kit. Upon blocking using 5% BSA for 1 hour, the sections were incubated in 1:100 diluted anti-Bcl2 antibody overnight at 4°C. Then, the sections were washed with PBS twice. Then we added biotin labeled secondary antibody to the slides and sequentially stained the slides with DAB and hematoxylin. The stained slides were recorded using the Nikon microscope. In TUNEL assay, the slides were stained with the terminal deoxynucleotidyl transferase-mediated dUTP-biotin nick-end labeling (TUNEL) method, using an apoptosis in situ detection kit (Wako Pure Chemical, Osaka, Japan). The FITC-labeled TUNEL-positive cells were imaged using the Nikon fluorescent microscope.

### 2.8. Plasmids and Transfection

Full-length lncRNA PVT1 sequences were cloned into a pcDNA 3.1 vector, termed pcDNA3.1-PVT1, and were selected along with neomycin for four weeks. LncRNA PVT1 shRNA oligos were synthesized, annealed, and then inserted into a lentiviral pLKO.1-Puro plasmid, termed shRNA-pLKO.1-Puro. HEK-293T cells were cotransfected with the above pLKO.1-Puro lentiviral vector, packaging vectors psPAX2, and pMD2.G envelope plasmid along with Lipofectamine2000™ reagent (Invitrogen). SGC-7901 cells were transfected with either the pcDNA3.1-PVT1 plasmid (PVT1) or the pcDNA3.1-vector (CTR), labeled as PVT1 overexpressing cells (positive control), and transfected with the shRNA- pLKO.1-Puro plasmid (shPVT1) or pLKO.1-Puro plasmid (NC), labeled as PVT1 silencing cells (negative control).

### 2.9. Cell Viability Assay

Cell viability was measured using the CCK8 method. In Brief, that entailed adding 10ul CCK-8 into the medium and mixed gently. The plate was subsequently incubated for 2 h and then the absorbance measurement was taken under a reference of 450nm.

### 2.10. Online Kaplan-Meier Plotter (KM-Plotter) Analysis

The KM-plotter is an online database (www.kmplot.com). It contains gene expression data and survival information downloaded from gene expression omnibus (GEO), European Genome-phenome Archive (EGA), and the Cancer Genome Atlas (TCGA). KM-plotter software can provide online survival analysis of GC patients with these available transcriptome and survival data according to different screening conditions [13].

### 2.11. Statistical Analysis

Statistical analyses were performed with the SPSS statistical package (SPSS, Inc., Chicago, IL, USA) and GraphPad Prism 6.0 (GraphPad Software). Experimental results were reported as a mean of at least three independent experiments conducted in triplicate. Two-tailed Student's t-test and Kaplan-Meier analyses were used as appropriate. P<0.05 were considered to be statistically significant.

## 3. Results

### 3.1. LncRNA PVT1 Can Regulate the Growth of GC

**Figure 1:**
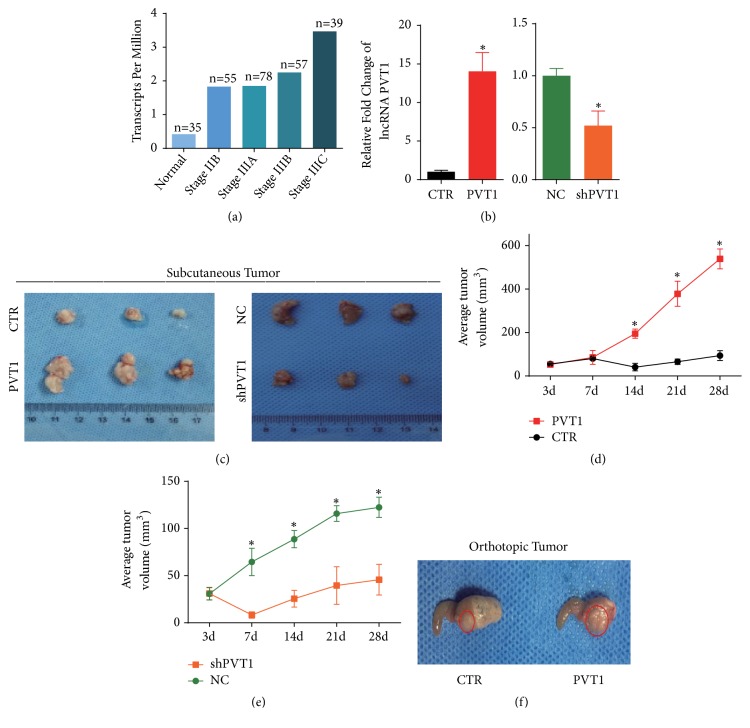
LncRNA PVT1 can regulate the growth of GC. (a) The expression of PVT1 in different stages of GC. A total of 431 samples were included in this dataset. These samples were divided into different subsets according to the tumor stages. The numbers refer to the number of people in the corresponding subsets. (b) This shows the validation of stably overexpressed and silenced PVT1 in SGC-7901 cells via qRT-PCR. PVT1 refers to PVT1 overexpression; CTR indicates the control group of PVT1; shPVT1 represents PVT1 silenced by shRNA; NC indicates the control of shPVT1. (c) This shows the subcutaneous tumors derived from SGC-7901 cells with overexpressed or silenced PVT1 (n=3 for each group). (d, e) This shows that the tumor volumes were measured at the indicated time. (f) This shows the orthotopic malignant tumor from SGC-7901 cells with overexpressed PVT1. Results were measured in triplicate in each experiment. *∗*P < 0.05.

First, we explored the expression of PVT1 in different stages of GC using the Cancer RNA-Seq Nexus (CRN; http://syslab4.nchu.edu.tw) so that we could access PVT1 gene expression data of GC patients from the TCGA stomach carcinoma RNA-Seq dataset [14]. As shown in Figure 1(a), the expression of PVT1 increased with cancer progression, compared to that of adjacent normal tissues. To explore the effects of PVT1 on GC, we stably overexpressed and stably silenced PVT1 expression in SGC-7901 cells (Figure 1(b)) and observed the influence of PVT1 on the tumorigenicity of gastric cancer cells in a mouse model. We noticed that the subcutaneous xenografts formed from PVT1-overexpressed GC cells had larger volume than xenografts formed from vector-control cells (Figures 1(c) left panel and 1(d)). Conversely, the subcutaneous xenografts formed from PVT1-silenced GC cells had smaller volume than vector-control tumors (Figures 1(c) right panel and 1(e)). An orthotopic mouse model was also established. As shown in Figure 1(f), the orthotopic xenograft formed from PVT1-overexpressed SGC-7901 cells also exhibited larger volume. Based on these results, we believe that PVT1 can regulate the growth of GC, and PVT1 may play a crucial role in the progression of GC.

### 3.2. LncRNA PVT1 Can Regulate the Apoptosis of GC

**Figure 2:**
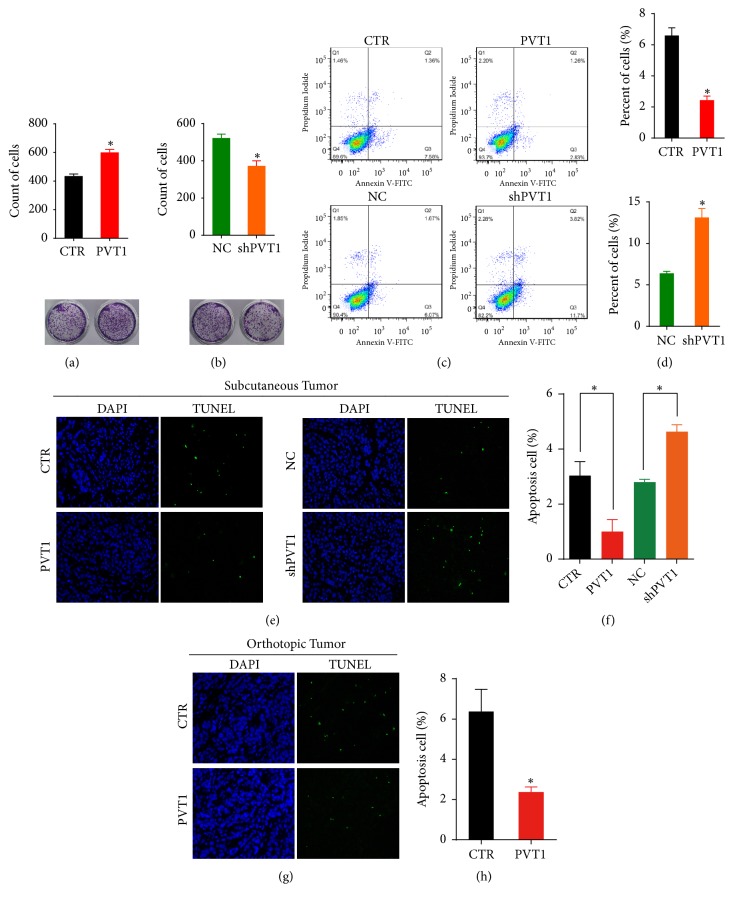
LncRNA PVT1 can regulate the apoptosis of GC. (a, b) This shows the clone formation assay of SGC-7901 with overexpressed and silenced PVT1. (c) The apoptosis cells were assessed using flow cytometry assay in PVT1 overexpressed and silenced SGC-7901 cells. (d) shows the quantitative results of the flow cytometry assay demonstrating the percentage of apoptotic cells. (e, g) TUNEL assay to detect apoptotic cells in xenografts tissues. The green cells indicate the TUNEL-positive apoptotic cells. The images of TUNEL-positive cells were captured by fluorescence microscope (400×). (f) Quantitative results of TUNEL assay of subcutaneous tumors tissues. (h) Quantitative results of TUNEL assay of orthotopic tumor tissues. All of the data shown represent the mean ± SD of three independent experiments. *∗*P < 0.05.

Next, we investigated the functions of PVT1 on GC cell behaviors. The clone formation assay showed that the count of clones formed by PVT1-overexpressed cells was significantly increased (Figure 2(a)), but significantly decreased by PVT1-silenced cells (Figure 2(b)). In the apoptosis analysis, upregulating PVT1 decreased the apoptosis of SGC-7901 cells; downregulating PVT1 increased the apoptosis of SGC-7901 cells (Figures 2(c) and 2(d)). To confirm the effects of PVT1 on apoptosis, TUNEL staining was conducted to investigate the apoptosis in the xenografts. Compared to the control xenograft, decreasing DNA fragmentation was observed in the subcutaneous xenograft formed by PVT1-overexpressed GC cells and increasing DNA fragmentation was observed from PVT1-silenced GC cells, respectively (Figures 2(e) and 2(f)). Similar results were also observed in the orthotopic xenograft (Figures 2(g) and 2(h)). Taken together, these results indicated that PVT1 can regulate the apoptosis of GC cells.

### 3.3. LncRNA PVT1 Regulates Apoptosis by Affecting Bcl2 Expression

**Figure 3:**
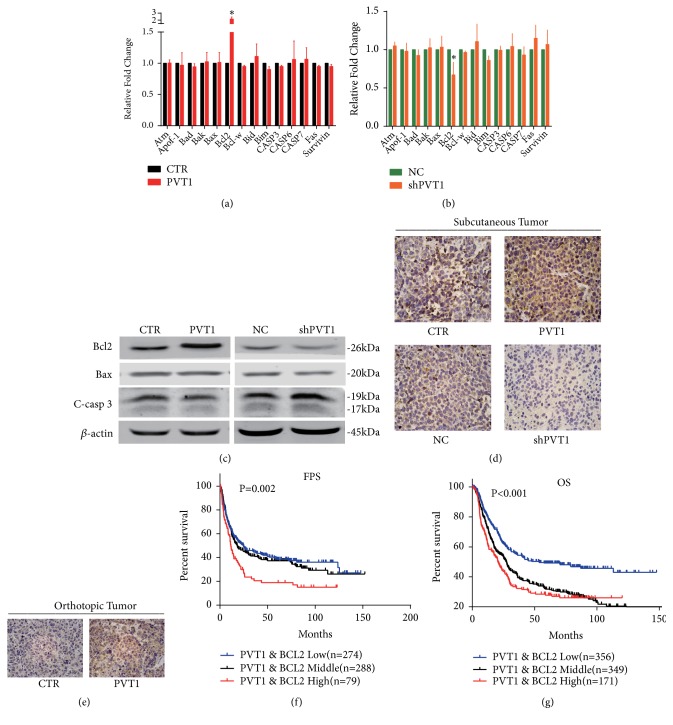
LncRNA PVT1 regulates apoptosis by affecting Bcl2 expression. (a, b) This shows the expression of common apoptosis related factors in PVT1 overexpressed or silenced SGC-7901 cells. Results for experiment were measured in triplicate. *∗*P < 0.05. (c) Western blot analysis showing protein levels of Bcl2, Bax, and cleaved caspase-3. *β*-actin was the internal control. (d) Representative IHC results of Bcl2 staining in subcutaneous tumor tissues from PVT1 overexpressed and silenced SGC-7901 cells. Results were measured in triplicate for each experiment (n=3 for each group). (e) Representative IHC results of Bcl2 staining in orthotopic tumor tissues. (f, g) GSE microarray (including GSE-14210, GSE-15459, GSE-22377, GSE-29272, GSE-51105, and GSE-62254) data were divided into three groups based on PVT1 and Bcl2 expression values; patients with both high expression of PVT1 and high expression of Bcl2 were divided into high group. Patients with both low expression of PVT1 and low expression of Bcl2 were divided into low group. The remaining patients were assigned to the middle group. Kaplan-Meier analysis was performed to compare their FPS and OS differences among the three groups.

**Figure 4:**
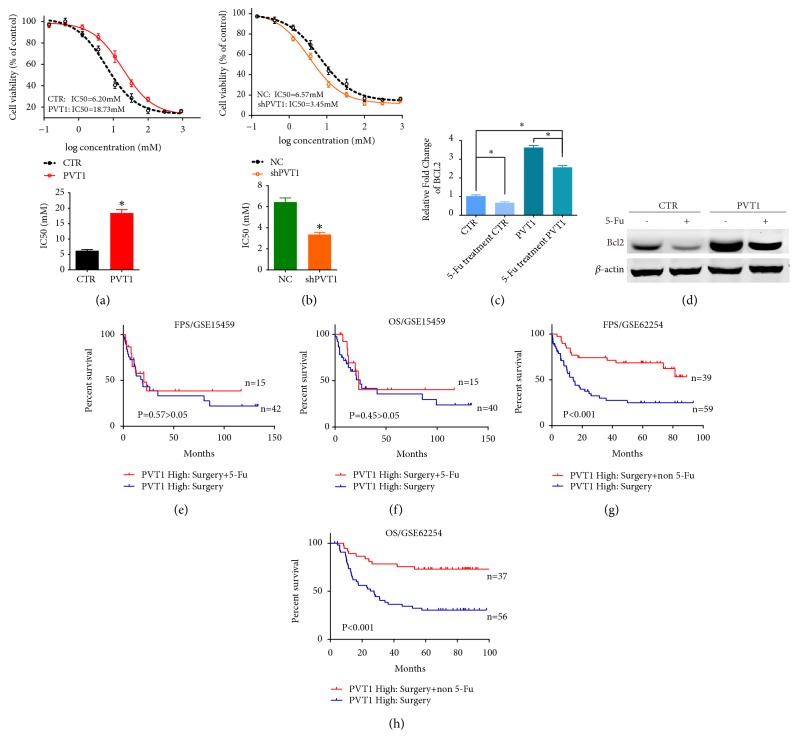
LncRNA PVT1 enhances drug-resistance of GC to 5-Fu. (a, b) Cell viability after 5-Fu treatment of PVT1 overexpressed and silenced SGC-7901 cells. (c) mRNA level of Bcl2 after 5-Fu treatment in SGC-7901 cells with or without overexpressed PVT1. Results were measured in triplicate for each experiment. *∗*P<0.05. (d) The alteration of Bcl2 protein after 5-Fu treatment in SGC-7901 cells with or without overexpressed PVT1; *β*-actin was the internal control. All of the data shown represent the mean ± SD of three independent experiments. (e, f) GSE15459 microarray data were divided into two groups based on the treatment, including high PVT1 level patients receiving both surgery and 5-Fu based adjuvant (PVT1 High: Surgery+5-Fu); high PVT1 level patients at stage III and IV receiving only surgery (PVT1 High: Surgery). Kaplan-Meier analysis was performed to compare their FPS and OS differences among the two groups. (g, h) The GSE62254 microarray data were divided into two groups, including high PVT1 level patients receiving both surgery and non-5-Fu adjuvant (PVT1 High: Surgery+non-5-Fu); high PVT1 level patients at stages III and IV receiving only surgery (PVT1 High: Surgery). *∗*P < 0.05.

To elucidate the underlying mechanism of PVT1 inducing apoptosis, we screened the common factors that may be related to this apoptosis process using the RT-PCR. The results showed that the Bcl2 mRNA was significantly increased when PVT1 was upregulated (Figure 3(a)) and significantly decreased when PVT1 was silenced (Figure 3(b)). Consistently, the following western blot indicated that the PVT1 can regulate the expression of Bcl2 protein but has no effect on the Bax protein. Beyond that, we noticed that the downstream apoptosis executor of Bcl2, cleaved caspase-3, was decreased when the PVT1 was overexpressed and increased when PVT1 was silenced (Figure 3(c)). To further verify the regulation of PVT1 on Bcl2, we evaluated the expression of Bcl2 protein in xenografts through the IHC assay. The IHC assay showed that the Bcl2 staining was evidently strengthened in subcutaneous xenografts formed from PVT1-overexpressed SGC-7901 cells but weakened in that from PVT1-silenced SGC-7901 cells (Figure 4(d)). Consistently, IHC showed Bcl2 staining was also strengthened when PVT1 was overexpressed in the orthotopic GC tumor (Figure 4(e)). In addition, Kaplan-Meier survival analysis in GEO datasets revealed that GC patients with both high level of PVT1 and Bcl2 suffered shortest first progression survival (FPS) (Figure 4(f)) and overall survival (OS) (Figure 4(g)). Collectively, those results demonstrated that the effects of PVT1 on apoptosis were achieved by regulating Bcl2. High levels of PVT1 combined with Bcl2 can predict poor prognosis in GC.

### 3.4. LncRNA PVT1 Enhances Drug-Resistance of GC to 5-Fu

It was reported that Bcl2 can determine the resistance of 5-Fu in carcinoma [15, 16]; hence, we supposed PVT1 may possibly enhance 5-fu resistance in GC via its promotion to Bcl2. To confirm this supposition, the IC50 values were estimated from growth inhibition curves. The results showed that, compared with their matched control group, the IC50 of 5-Fu was significantly increased when PVT1 was overexpressed (Figure 4(a)), but significantly decreased when PVT1 was silenced (Figure 4(b)). The following RT-PCR and western blotting assay showed that the 5-Fu treatment can decrease Bcl2 mRNA and protein of GC cells. Although 5-Fu had an inhibitory effect on Bcl2 in PVT1-overexpressed SGC-7901 cells, the same dose of 5-Fu treatment could not offset the promoting effect of PVT1 on Bcl2 and the level of Bcl2 after 5-Fu treatment was still significantly high compared to the norm (Figures 4(c) and 4(d)).

Next, we analyzed the prognosis of 5-Fu treatment in GC with high PVT1 expression using the follow-up data from GEO. The KM-plotter analysis compared the FPS and OS of GC patients in later stages (stage III or IV), whereby high PVT1 expression patients only received gastrectomy and patients with high PVT1 expression received both gastrectomy and 5-Fu based adjuvant (or other non-5-fu based drug). Analyses showed that, in patients with high PVT1 expression, the addition of 5-Fu based adjuvant did not improve the FPS (Figure 2(e)) and OS (Figure 2(f)) of GC patients; however, the adjuvant without 5-Fu improved the FPS (Figure 2(g)) and OS (Figure 2(h)) of GC patients significantly.

Taken together, these findings demonstrated that PVT1 can enhance the resistance of GC to 5-Fu. The therapeutic effect of 5-Fu based adjuvant is limited in high PVT1 expression GC patients.

## 4. Discussion

A growing amount of evidence indicates that lncRNA can play an important role in regulating the apoptosis in malignant tumors. It was reported that some lncRNAs are negative regulators of apoptosis in different types of cancer. For example, lncRNA GAS5 can inhibit apoptosis of non-small-cell lung cancer cells, through upregulating P53 expression and downregulating transcription factor E2F1 expression [17]. LncRNA AFAP1-AS1 was found to be hypomethylated and overexpressed in esophageal cancer [18]. LncRNA PlncRNA-1 was upregulated in prostate cancer samples and cell lines, so it can work as an inhibitor of apoptosis through promoting the cleavage of PARP-1, a key component of the DNA damage response [19]. For malignant tumors, a large percentage of cell loss was due to apoptosis, especially in spontaneously regressing tumors and in tumors treated with cytotoxic anticancer agents. It was suggested that apoptosis is linked to the high rate of cell loss in malignant tumors and could promote the progression of the tumor [11]. In the present study, we confirmed that PVT1 can inhibit the apoptosis of GC; although this inhibition is modest, the inhibitory effect on apoptosis may be one of the pathways for PVT1 to promote the progression of GC.

There are mainly two independent apoptosis signal cascades. The extrinsic apoptosis is always activated by the specific death receptors on the cellular membrane [20]. The intrinsic pathway is usually caused by DNA damage or growth factor withdrawal, which can promote the release of cytochrome C and other proteins from the intermembranous space of the mitochondria [21]. The control and regulation of intrinsic apoptotic mitochondrial events occur through members of the Bcl2 family of proteins, which comprises both antiapoptotic proteins such as Bcl-2 and proapoptotic proteins such as Bax. Bcl2 is located primarily in the outer mitochondrial membrane and blocks apoptosis by preventing cytochrome c release from the mitochondria, as well as by inhibiting caspase-3 activity. In contrast, proapoptosis protein Bax can release cytochrome c from the mitochondria to promote apoptosis. High Bcl2/Bax ratio is regarded as a crucial factor of cellular resistance to apoptosis [22–25]. In this study, we revealed that overexpressing PVT1 can significantly increase the Bcl2/Bax ratio and decrease the expression of downstream cleaved caspase-3 (Figure 3(c)). This intrinsic mechanism may account for the inhibition of PVT1 on apoptosis.

In addition to apoptosis inhibition, the Bcl2 protein is correlated with cancer resistance to chemotherapeutic drugs [26]. Contrary to protecting cancer cells from drug induced cell cycle arrest, Bcl2 can prolong their survival during this period; hence, proliferation resumes upon withdrawal of the drug. It was reported that 5-Fu can decrease the expression of Bcl2 [22], and in our study, we observed the same results on GC. However, in PVT1-overexpressed GC cells, the expression of Bcl2 after 5-Fu treatment was still much higher than control cells (Figures 4(c) and 4(d)), which indicated that the Bcl2 activated by PVT1 was involved in drug-resistance to 5-Fu.

As a basic chemotherapeutic drug, 5-Fu is widely used in the treatment of a variety of cancers, including gastrointestinal cancer, breast cancers, and neck cancers. By inhibiting essential biosynthetic processes and disrupting DNA and RNA synthesis, 5-FU treatment can significantly decrease the recurrence rate of tumors and improve PFS and OS of patients [27]. In GC, 5-Fu is the core of anticancer agents. 5-FU-based chemotherapy was recommended as the first-line treatment in adjuvant therapy, and in some cases, 5-fluorouracil derivative has been delivered as a single agent according to the recent Japanese guidelines. As the most commonly used anticancer agent in clinic, 5-FU-based agents have always been administrated as the first choice treatment modality in advanced GC but without sensitivity screening. Nonetheless, response rates for 5-FU chemotherapy for GC are only 29.5% [28, 29]. Under these circumstances, the 5-Fu based chemotherapy strategy would miss the optimal period for adjuvant therapy and cause a waste of medical resources and hurt the economy when it comes to patients with initial 5-Fu resistance. Thus, it is imperative to develop an effective way to predict the resistance to 5-Fu chemotherapy and then formulate more reasonable and effective treatment plans. In our study, we elucidated that high PVT1 can cause a resistance to 5-Fu (Figures 4(a) and 4(b)). Survival analyses indicated that, in GC patients with high levels, the addition of 5-Fu based adjuvant after gastrectomy did not bring additional benefit in prognosis than compared to only gastrectomy (Figures 2(e) and 2(f)). On the contrary, chemotherapy without 5-Fu after gastrectomy could benefit these patients significantly (Figures 2(g) and 2(h)). We verified the resistance to 5-Fu induced by PVT1, and the study implicated that the relative level of PVT1 can serve as an indicator to predict the 5-Fu resistance of GC and a reference to formulate suitable treatment plan.

In summary, our results indicate that lncRNA PVT1 can activate the expression of antiapoptosis factor Bcl2. This may be one of the pathways for PVT1 to promote the progression of GC. In high PVT1 expression GC patients, PVT1 enhances the drug-resistance of GC to 5-Fu; thus a non-5-FU based chemotherapy regimen may be a better treatment option.

## Data Availability

The survival analysis data used to support the findings of this study were supplied by Kaplan-Meier Plotter (http://kmplot.com/analysis/).

## Supplementary Materials

Supplementary Materials Table S1: this table lists the premier of apoptosis related factors in real-time PCR screening.
