# Supplementary material for: LncRNA PVT1 Mediates Antiapoptosis and 5-Fluorouracil Resistance via Increasing Bcl2 Expression in Gastric Cancer
Source: J Oncol. 2019 May 8;2019:9325407. doi: 10.1155/2019/9325407 (PMC6530232; doi:10.1155/2019/9325407)
Supplement: Supplementary Materials — Table S1: this table lists the premier of apoptosis related factors in real-time PCR screening. [file 9325407.f1.docx]

**Supplementary Table**

**Table S1.** The premier of apoptosis related factors in screening

| Premier | Sequences |
| --- | --- |
| Apaf-1 foreward | TGAGCCCACTCAACAGCAAA |
| Apaf-1 reverse | TGTCCTTACACTGGAAGAAGAGA |
| ATM foreward | TCTCAGCAACAGTGGTTAGAATTG |
| ATM reverse | CCGTCAGTCTGAGAACAGCA |
| Bad foreward | CTTGTCCTCACAGCCCAGAG |
| Bad reverse | CCCCAGCGCCTCCATGAT |
| Bak foreward | CCATTCCTGGAAACTGGGCT |
| Bak reverse | GACGGGATCAGCCTGCC |
| Bax foreward | CAAACTGGTGCTCAAGGCCC |
| Bax reverse | GAGACAGGGACATCAGTCGC |
| Bcl-w foreward | CTTTGTCTTTGGGGCTGCAC |
| Bcl-w reverse | CTGTGAACTCCGCCCAGC |
| Bid foreward | GCCATAAGGAGGAAGCGGG |
| Bid reverse | ACCGTTGTTGACCTCACAGT |
| Bim foreward | GTATTCGGTTCGCTGCGTTC |
| Bim reverse | CCTCATGGAAGCTTGTGGCT |
| Caspase 3 foreward | CGGCGCTCTGGTTTTCGTTA |
| Caspase 3 reverse | CCGAGATGTCATTCCAGTGCT |
| Caspase 6 foreward | GGACCACAGGAGGAGAGGAAT |
| Caspase 6 reverse | GTGGCTAACAGTTGACACCTCA |
| Caspase 7 foreward | GTGGGAACGATGGCAGATGA |
| Caspase 7 reverse | GAGGGACGGTACAAACGAGG |
| Fas foreward | GTGACCCTTGCACCAAATGT |
| Fas reverse | AGACAAAGCCACCCCAAGTT |
| Survivin foreward | AGGACCACCGCATCTCTACA |
| Survivin reverse | TTTCCTTTGCATGGGGTCG |
